# Peptide-Based pH-Responsive MRI-CEST Agents: In Vivo Comparison between a Selected Pentapeptide and the Established Iopamidol Reference in Tumor pH Mapping Ability

**DOI:** 10.1021/cbmi.5c00150

**Published:** 2026-01-01

**Authors:** Enrico Gallo, Antonella Carella, Francesco Gammaraccio, Marco Salvatore, Antonella Accardo, Dario Livio Longo, Silvio Aime

**Affiliations:** † 591458 IRCCS SYNLAB SDN, via G. Ferraris 144, 80146 Napoli, Italy; ‡ Istituto di Biostrutture e Bioimmagini, 9327 Consiglio Nazionale delle Ricerche, Sede Secondaria di Torino, via Nizza 52, 10126 Torino, Italy; § Department of Pharmacy and Interuniversity Research Centre on Bioactive Peptides (CIRPeB) “Carlo Pedone”, 9307 University of Naples Federico II, via D. Montesano 49, 80131 Napoli, Italy

**Keywords:** peptides, MRI, CEST, tumor, contrast agents

## Abstract

Magnetic resonance imaging based on chemical exchange saturation transfer (MRI-CEST) has emerged as a powerful imaging technique for mapping physiological parameters, such as tissue pH, with high spatial resolution. This study explores the pH-responsive performance of a novel peptide-based CEST agent, selected among several candidates, when compared with the established contrast agent iopamidol. The selected hLys containing pentapeptide showed enhanced sensitivity and biocompatibility while maintaining an excellent CEST response. In vivo MRI studies further assessed its applicability for tumor pH imaging. The results demonstrate that the selected peptide-based agent displays good CEST contrast variations in response to pH changes, highlighting its potential as a pH-responsive CEST agent. The assessed pH values were very similar to those obtained upon the administration of iopamidol, a well-established pH-CEST agent. Notably, this result was obtained by administering a mass dose of contrast agent that is about 8-fold less than that used in the case of iopamidol. These findings pave the way for the development of peptide-derived MRI probes for noninvasive tumor microenvironment assessment.

## Graphical abstract

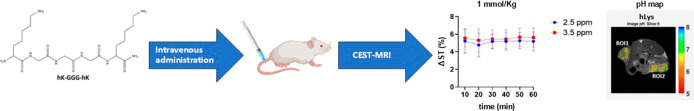

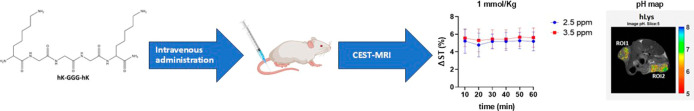

## Introduction

MRI has gained a leading position among in vivo imaging modalities due to its superb spatial and temporal resolution. The attainable diagnostic information is further enhanced through the use of suitable contrast agents (CAs), which provide additional physiologic information beyond anatomical details.^1^ Clinically used agents are mostly represented by paramagnetic complexes able to enhance the water proton relaxation rate in the regions where they distribute. Currently, approximately 40% of MRI scans acquired at clinical settings employ Gd-based CAs, as they report abnormalities in vasculature leakiness and tissue perfusion in the presence of tumors.^2^ However, these relaxation-enhancing agents do not provide insight into the metabolic activity of tumor cells. To address this limitation, an emerging MRI technique has been developed that modulates contrast based on the selective transfer of saturated magnetization to the water resonance.^3^ This method, known as chemical exchange saturation transfer (CEST), involves applying a radiofrequency (rf) irradiation at the chemical shift of the exchanging proton pool, leading to a selective transfer of saturated magnetization to the water resonance.^4^ The water signal decreases in intensity as a function of the concentration of exchanging protons, the exchange rate, the intensity of the rf field, and the relaxation time of water protons.^5^ CEST is a frequency encoding approach that reports on the specific contribution from a given exchanging pool of protons only if the frequency of the applied rf field matches the chemical shift of the mobile protons’ resonance. Both endogenous molecules (e.g., amino acids, peptides, proteins, polysaccharides) and exogenous systems can act as CEST CAs.^6–11^ Furthermore, when multiple mobile protons with distinct functional groups are present on the same molecule, their different saturation transfer (ST) effects can be exploited in the setup of ratiometric approaches for specific assessments.^12,13^ This approach eliminates the need for precise knowledge of the agent’s concentration and enables the extraction of valuable information about the determinants of the ongoing exchange process. Among the parameters accessible via such a ratiometric approach, pH is by far the most extensively investigated one.^14^

In the context of tumors, the pH of the extracellular matrix (ECM) serves as a critical metabolic hallmark that has garnered significant attention.^15,16^ In most solid tumors, the ECM pH is shifted toward acidic values due to the enhanced glycolytic metabolism, which produces high levels of lactic acid that are promptly released from the intracellular compartment.^17,18^

Additionally, further proton production can result from the activity of carbonic anhydrase on the outer surface of tumor cells.^19,20^ This enzyme generates bicarbonate ions, which are transported into the cell via specific transporters, contributing to alkalinization of the intracellular compartment. Overall, the decreased pH in the ECM is considered an excellent hallmark of tumor metabolism and a marker of its aggressiveness.^21,22^

Several MRI-CEST approaches have been employed to spatially resolve tissue variations in pH, including endogenous molecules, para- and dia-CEST agents.^23,24^ In the case of endogenous systems, it was early shown that amide proton transfer (APT) can measure differences in pH.^25^ The chemical exchange between the amide protons and the bulk water is base-catalyzed, so that the exchange rate decreases with decreasing pH. However, the generated contrast depends on many parameters that compromise the measurement of pH. For this reason, the pH-weighted APT image is essentially used to measure relative changes in pH, but not absolute pH values.^26,27^ Many para-CEST agents based on the exploitation of the ratiometric procedure, based on the frequency-selective labeling of the mobile proton pools, were reported.^28^ The first series dealt with Ln-DOTAMGly ( Ln = Pr, Nd, Eu).^29^ The CEST effect from an amide is pH-responsive, while CEST from the metal-bound water is pH-unresponsive. Unfortunately, owing to the fast exchange rate of metal-bound water at physiologic temperature, high saturation power is required, thus limiting their interest to in vivo translation.^30^ Therefore, instead of detecting the CEST effect from a metal-bound water, the CEST effects of hydroxyl groups in Yb-HPDO3A, an analogue to Food and Drug Administration (FDA) approved ProHance (Gd-HPDO3A), were proposed.^31^ The ratio of hydroxyl CEST effects arises from the two isomeric forms of this species and was linearly correlated within the pH range of interest. A further improvement in this class of para-CEST pH reporting agents was brought with Yb-DO3AoAA, which has an aryl amine and amide functionalities.^32^

In the field of dia-CEST agents, many pH-dependent systems have been investigated, exploiting the pH-dependence of the prototropic exchange of amide, amine, and hydroxyl functionalities. This approach included the study of simple peptides, polypeptides, and dendrimers.^8,33–41^

Peptides have been proposed as promising candidates for CEST-based imaging agents.^8^ A potential approach in MRI-CEST involves designing functional peptides that alter their contrast in response to environmental factors, such as enzymatic activity or pH changes. For example, phosphorylation of synthetic peptides like kemptide^42^ and human protamine-1 (hPRM1)^43^ significantly reduces CEST contrast by slowing proton exchange rates. These examples demonstrate that the presence of a negative charge near an exchangeable proton can significantly influence the CEST contrast by reducing the proton exchange rate. Although this effect can be utilized in the design of peptide-based sensors, a more favorable approach would be to enhance the exchange rate to optimize the CEST contrast.

The system that encountered the most attention as a pH reporter is iopamidol, a widely used CA for X-ray computed tomography (CT/X-ray) imaging. This agent has two amide functionalities that display a different absorption frequency as well as a different pH dependence of their prototropic exchange.^44,45^ On this basis, a ratiometric method was established to assess the absolute pH of the extracellular microenvironment in which the agent distributes.^46–49^ An issue related to the use of iopamidol is the large amount of the compound that needs to be administered. The largest part of the injected mass is obviously not contributing at all to the intended use as a pH reporter. Thus, we deemed it of interest to investigate whether simple peptides containing amide and amine functionalities as a source of exchangeable protons may represent useful dia-CEST systems when applied at molar concentrations like those used for iopamidol but involving much lower amounts in terms of the corresponding weights of administered CA.

## Materials and Methods

N^α^-Fmoc-protected amino acids, Rink amide MBHA (4-methylbenzhydrylamine) resin, and all reagents for coupling reactions were purchased from Calbiochem-Novabiochem (Läufelfingen, Switzerland). Peptide synthesis solvents and other chemical products were of reagent grade and were obtained from Merck (Milan, Italy), LabScan (Stillorgan, Dublin, Ireland), or Fluka (Buchs, Switzerland). All chemicals were used without further purification unless otherwise specified.

### Solid Phase Peptide Synthesis and Purification

Peptide sequences [Dap-GGG-Dap (Dap), Dab-GGG-Dab (Dab), Orn-GGG-Orn (Orn), K-GGG-K (Lys), hK-GGG-hK (hLys), K­(CH_3_)-GGG-K­(CH_3_) (LysCH_3_), and H-GGG-H (His)] were synthesized following standard solid-phase peptide synthesis (SPPS) 9-fluorenylmethoxycarbonyl (Fmoc) protocols.^50^ Briefly, Rink amide MBHA resin (substitution of 0.70 mmol g^–1^) was used as the solid support and swelled in N,N-dimethylformamide (DMF) for 45 min. The Fmoc protecting group was removed using 20% v/v piperidine in DMF for two cycles of 8 min each, followed by thorough washing with DMF. Subsequently, each amino acid was coupled by treating the resin for 45 min with a solution containing 2 equiv of the corresponding Fmoc-protected amino acid dissolved in DMF. The reaction was activated using 2 equiv of 1-hydroxybenzotriazole (HOBt), 2 equiv of benzotriazol-1-yl-oxytris-pyrrolidino-phosphonium (PyBOP), and 4 equiv of diisopropylethylamine (DIPEA). This deprotection and coupling cycle was repeated until the desired peptide sequences were achieved. At the end of the synthesis, the peptides were removed from the solid support while simultaneously removing all protecting groups using a trifluoroacetic acid (TFA)/triisopropylsilane (TIS)/H_2_O solution (92.5:5:2.5 v/v/v) under stirring at room temperature for 3 h. Peptides were then precipitated in cold ether and lyophilized three times. Purification of crude peptides was performed via preparative reversed-phase high-performance liquid chromatography (RP-HPLC), using a Phenomenex (Torrance, CA, USA) C18 column and a Shimadzu LC8 HPLC system (Shimadzu Corporation, Kyoto, Japan) furnished with a UV Lambda-Max Model 481 detector. The elution solvents were H_2_O/0.1% TFA (A) and CH_3_CN/0.1% TFA (B) from 0% to 20% over 20 min at a flow rate of 20 mL·min^–1^.

Pure peptides were identified through liquid chromatography–mass spectrometry (LC–MS) using an LTQ XL Linear Ion Trap Mass Spectrometer with an electrospray ionization source (ESI) (Figure S1). ESI source parameters: sheath gas flow rate (arb.) = 8; spray voltage = 3.50 kV; spray current = 1.20 μA; capillary temperature = 275 °C; capillary voltage = 31 V; tube lens = 100 V; and through analytical RP-HPLC performed by using Finnigan Surveyor MSQ single quadrupole electrospray ionization (Finnigan/Thermo Electron Corporation San Jose, CA, USA), with a C18-Phenomenex column eluting with H_2_O/0.1% TFA (A) and CH_3_CN/0.1% TFA (B) from 0% to 10% over 20 min at a flow rate of 1 mL min^–1^. After the purification, peptides were obtained in high yield (>80%) and purity (>95%).

The analytical results, including retention times from RP-HPLC and mass/charge (*m*/*z*) values from ESI spectrometry and the p*K*_a_ values, obtained by applying the ChemDraw software, are summarized in Table 1. These data validate the successful synthesis and characterization of the peptides, ensuring their suitability for subsequent CEST performance evaluation.

**Table 1:** Formula, Calculated and Experimentally Found Molecular Weight (MW), Retention Time, and Theoretical *p*K_a_ of the Investigated Peptides

peptide	acronym	formula	MW_calc._(a.m.u.)	MW_deter._(a.m.u.)	retention time (min)	p*K*_a_ calc.
Dap-GGG-Dap	Dap	C_12_H_24_N_8_O_5_	360.4	361.3	6.2	3.69
						7.70
						8.52
Dab-GGG-Dab	Dab	C_14_H_28_N_8_O_5_	388.4	389.3	5.8	5.58
						9.10
						9.38
Orn-GGG-Orn	Orn	C_16_H_32_N_8_O_5_	416.5	417.3	5.4	6.48
						9.71
						9.91
K-GGG-K	Lys	C_18_H_36_N_8_O_5_	444.5	445.4	4.9	6.92
						10.01
						10.15
hK-GGG-hK	hLys	C_20_H_40_N_8_O_5_	472.6	473.4	4.6	7.27
						10.19
						10.15
K(CH_3_)-GGG-K(CH_3_)	LysCH_3_	C_20_H_40_N_8_O_5_	472.6	473.4	4.6	6.97
						9.93
						10.07
H-GGG-H	His	C_18_H_26_N_10_O_5_	462.5	463.3	4.9	7.07

### Phantoms

Phantoms containing the peptides whose structures are reported in Figure 1 (Dap, Dab, Orn, Lys, hLys, LysCH_3_, and His) were prepared for in vitro analysis. The phantoms consisted of a 50 mL Falcon conical tube (diameter of 30 mm and length of 115 mm) filled with water and containing six vials of 10 mM peptide solution dissolved in phosphate-buffered saline 1× (PBS), with pH titrated between 6 and 8. In addition, MR CEST images of phantoms containing vials of each peptide at concentrations of 5 and 2.5 mM and at pH 7.4 were acquired.

**Figure 1:**
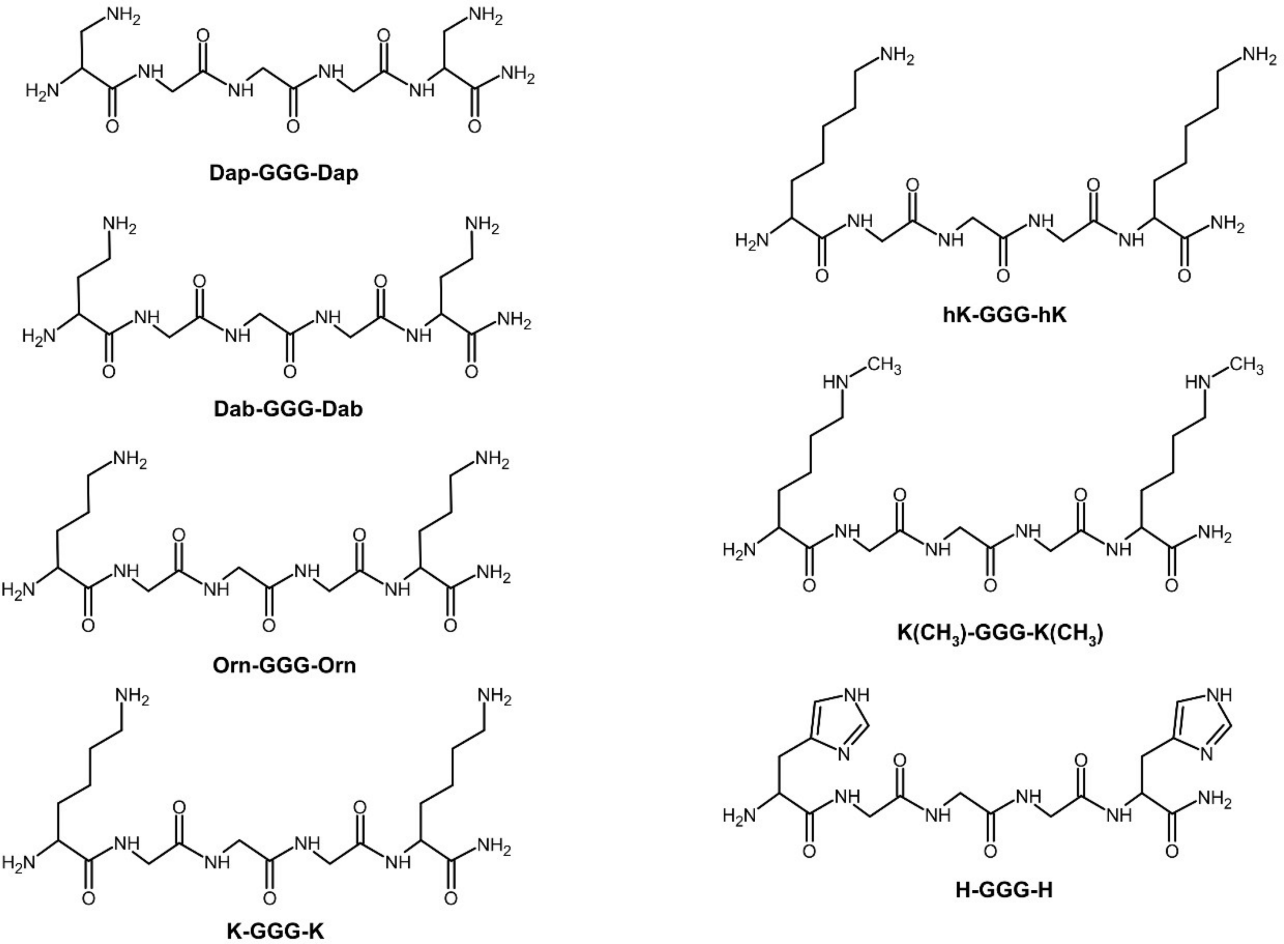
Chemical structures of the investigated peptides: Dap-GGG-Dap (Dap), Dab-GGG-Dab (Dab), Orn-GGG-Orn (Orn), K-GGG-K (Lys), hK-GGG-hK (hLys), K­(CH_3_)-GGG-K­(CH_3_) (LysCH_3_), and H-GGG-H (His).

For temperature-controlled MR imaging, the phantom was heated in a water bath for approximately 1 h and then rapidly inserted into the MRI magnet and maintained at 33 °C using hot air controlled by a temperature sensor.

### MRI In Vitro Experiments

MRI scans were obtained using a 7T Avance Neo Microimaging Bruker MRI scanner (Bruker Biospin, Ettlingen, Germany), which was fitted with a 30 mm 1H quadrature coil and with images acquired at both room temperature and at 33 °C. After the scout image acquisition, T_2w_ anatomical images were acquired using a RARE sequence (TR = 4 s, TE = 34 ms, NA = 1, slice thickness = 1.5 mm, field of view = 30 × 30 mm^2^, matrix size = 96 × 96). CEST images were acquired with a Fast-Spin–Echo acquisition sequence (TE = 3.77 ms, TR = 12 s, slice thickness = 1.5 mm, number of slices = 1, Field of View = 30 × 30 mm^2^, matrix size = 128 × 128) by applying a continuous-wave saturation pulse (power: 1.5 μT; duration: 5 s) sampling 183 offsets in the range ± 10 ppm. The total acquisition time for each CEST volume was ca. 36 min.

### Peptide Serum Stability

The hLys powder was dissolved in double-distilled water in order to prepare a 1 mM solution. This solution was mixed 1:1 (v/v) with human serum (Sigma-Aldrich, USA) and incubated at 37 °C with gentle shaking. Then, 150 μL aliquots were taken at different time points, and protease activity was stopped with 20 μL of 15% (v/v, in H_2_O) trichloroacetic acid (TCA). After 30 min at 4 °C, the samples were centrifuged at 13,000*g* for 12 min to remove serum proteins. The supernatants were analyzed by analytical RP-HPLC and LC–MS, as previously described (Figure S2). The percent of intact peptide was calculated by peak integration using Winchrom software (GBC Scientific), and expressed as a percent of the amount at *t*_0_. Data were fitted to a one-phase exponential decay model using GraphPad Prism version 7 to estimate the peptide half-time (*t*_1/2_).

### Cell Culture

A murine mammary carcinoma 4T1 cell line was used for the in vivo experiments. 4T1 cell line was purchased from the American Type Culture Collection (ATCC LGS standards, Sesto San Giovanni, Italy). Cells were grown in RPMI-1640 medium, supplemented with 10% fetal bovine serum (FBS), 100 U/mL penicillin, 100 μg/mL streptomycin (Pen/Strep), and 2 mM l-glutamine. Cells were cultured in a humidified incubator with 5% CO_2_ at 37 °C and 60% relative humidity.

### MRI In Vivo Experiments

All animal procedures and husbandry were performed in accordance with the University of Torino Ethical Committee and European guidelines under Directive 2010/63. Eight-week-old male BALB/C mice (*n* = 6) were bred at the animal facility of the Department of Molecular Biotechnology and Health Sciences, University of Torino, Italy. Mice were inoculated subcutaneously with 1 × 10^6^ 4T1 cells on both flanks. When the tumors were approximately 40 mm^3^, mice were anesthetized by injecting a mixture of xylazine 5 mg/kg (Rompun, Bayer, Milan, Italy) and tiletamine/zolazepam 20 mg/kg (Zoletil 100, Virbac, Milan, Italy) for the acquisitions. MR images were acquired on a 7T Avance Neo Microimaging Bruker scanner (Bruker Biospin, Ettlingen, Germany) equipped with a 30 mm 1H quadrature coil. Breath rate was monitored by an air pillow placed below the animal (SA Instruments, Stony Brook, NY, USA), and the tail vein was cannulated with a catheter through a 27-gauge needle. The same mice were imaged twice: first for evaluating the CEST contrast properties of the hLys peptide (*n* = 3 mice) and 2 days after for comparing the pH mapping (*n* = 3 mice) between hLys and iopamidol upon consecutive administration of the two CAs.

After the scout image acquisition, T_2w_ anatomical images were acquired using a RARE sequence (TR = 4 s, TE = 34 ms, NA = 1, slice thickness = 1.5 mm, field of view = 30 × 30 mm^2^, matrix size = 256 × 256). CEST images were acquired before and after the i.v. injection of hLys (dose = 1 mmol/kg body weight) using a fast multislice single-shot centric-encoding sequence preceded by a continuous-wave saturation pulse (power: 1.5 μT; duration: 5 s) covering the whole tumor (TE = 3.77 ms, TR = 12 s, slice thickness = 1.5 mm, number of slices = 8, Field of View = 30 × 30 mm^2^, matrix size = 128 × 128).^51^ The total acquisition time for each CEST volume was 10 min. In the first cohort of mice, the CEST acquisitions were repeated for approximately 60 min after CA injection in order to evaluate the ST% contrast over time. In the second cohort, 30 min after the i.v. injection of the hLys peptide, additional MRI-CEST images were acquired before and after i.v. injection of 4 g of iodine/kg of b.w. of iopamidol (Bracco Imaging SpA, Colleretto Giacosa, Italy) into the tail vein.

### CEST Imaging Analysis

All CEST images were processed using a custom script developed in MATLAB (The Mathworks, Inc., Natick, MA, USA). The Z-spectra were interpolated voxel by voxel by using cubic smoothing splines for B0 correction, and the ST% efficiency was calculated on the corresponding frequency offset for the different peptides by asymmetry analysis.^52^ For the in vivo studies, to minimize the impact of endogenous contributions, difference contrast maps (ΔST%) were generated by subtracting the ST contrast after CA administration from the preinjection ST contrast for each voxel (at 2.5 and 3.5 ppm for hLys and at 4.2 and 5.5 ppm for Iopamidol, respectively).^53^ For both hLys and iopamidol injection, tumor pHe values were calculated in vivo using a ratiometric approach between the two different offsets for each pH-responsive CA, based on the previously calculated pH calibration curve (for Iopamidol^53^) and on the calculated calibration curve for hLys (Figure S3) based on the following equation:

pH(hLys)=0.139×(ST_ratio)^2−0.953×(ST_ratio)+7.501

where ST_ratio is the ratio of the CEST contrast between 2.5 and 3.5 ppm for the hLys.

The resulting pH maps were overlaid onto the anatomical reference image.

## Results and Discussion

### Design and Synthesis of the Peptides

The aim of the present study was to evaluate the CEST properties of a set of pentapeptides designed to have a good number of exchangeable protons on different functionalities in order to set up a ratiometric method to map pH. Most of the systems rely on a design that focuses on the presence of amide groups and amine (primary and secondary). Each peptide is composed of a common central Gly-Gly-Gly (GGG) motif, with terminal amino acids bearing an amine functionality on their side chains, differing in the length of the methylene side chain (respectively −CH_2_–NH_2_ for Dap; −CH_2_–CH_2_–NH_2_ for Dab; −CH_2_CH_2_CH_2_–NH_2_ for Orn; −CH_2_CH_2_CH_2_CH_2_–NH_2_ for Lys, −CH_2_CH_2_CH_2_CH_2_CH_2_–NH_2_ for hLys and −CH_2_CH_2_CH_2_CH_2_–NH–CH_3_ for LysCH_3_. One peptide contains imidazole moieties as a source of exchanging protons (H-GGG-H or His)^54^ (Figure 1).

The substitution of lysine with analogues bearing different lengths of the aliphatic chain was intended to systematically affect the spatial and electronic properties of the side chain, thereby enabling the setup of intramolecular H-bonding that would affect the proton exchange rate and, in turn, the overall CEST performance of the given peptide.^55^ Besides the design of efficient imaging pH reporters, the CEST characterization of the modulation of the exchange dynamics may offer useful insights into the use of these peptides for future biomedical applications.

All the peptides in this study were synthesized using SPPS following standard Fmoc-based protocols.^29^ This method was chosen for its efficiency and reliability in assembling peptides with high purity and precise sequence control.^56,57^ After synthesis, the peptides were thoroughly characterized to confirm their identity and purity (Figure S1). Characterization was performed through RP-HPLC to assess the purity of the products and ensure the proper separation of the synthesized compounds. ESI mass spectrometry was employed to confirm the molecular weights and structural integrity of the peptides, providing detailed insights into the successful incorporation of amino acid residues and the absence of unexpected modifications or side products. The analytical results, including retention times from RP-HPLC and mass/charge (*m*/*z*) values from ESI spectrometry, are summarized in Table 1.

### In Vitro MRI-CEST Results

The CEST contrast profiles and the corresponding ratiometric values of all of the investigated peptides were compared by measuring the ST effect at 2.5 and 3.5 ppm, respectively. In principle, each ST profile represents a sort of fingerprint that reflects the pH dependence of each peptide. However, it appeared more practicable to go with the assessment of the ST ratio at the two applied irradiation frequencies. The normalized Z-spectra and corresponding ST% values calculated from the MTRasym curves clearly showed the presence of two maxima at saturation-frequency offsets of approximately 2.5 and 3.5 ppm. These absorptions are ascribed to amine (2.5 ppm) and amide (3.5 ppm) groups, respectively.

At 21 °C and pH 7.4, whereas the His, Dap, Dab, Orn, and Lys peptides showed comparable CEST contrast of 2% (at 2.5 ppm, Figure 2A) and of 5% (at 3.5 ppm, Figure 2B), hLys and LysCH3 showed significantly higher CEST contrast values (6% and 10% at 2.5 and 3.5 ppm, respectively). When evaluating the pH dependence of the ratiometric values, it was confirmed that the Lys- and hLys-containing peptides own the highest pH sensitivity over the entire range of pH from 6.0 to 8.0 (Figure 2C).

**Figure 2:**
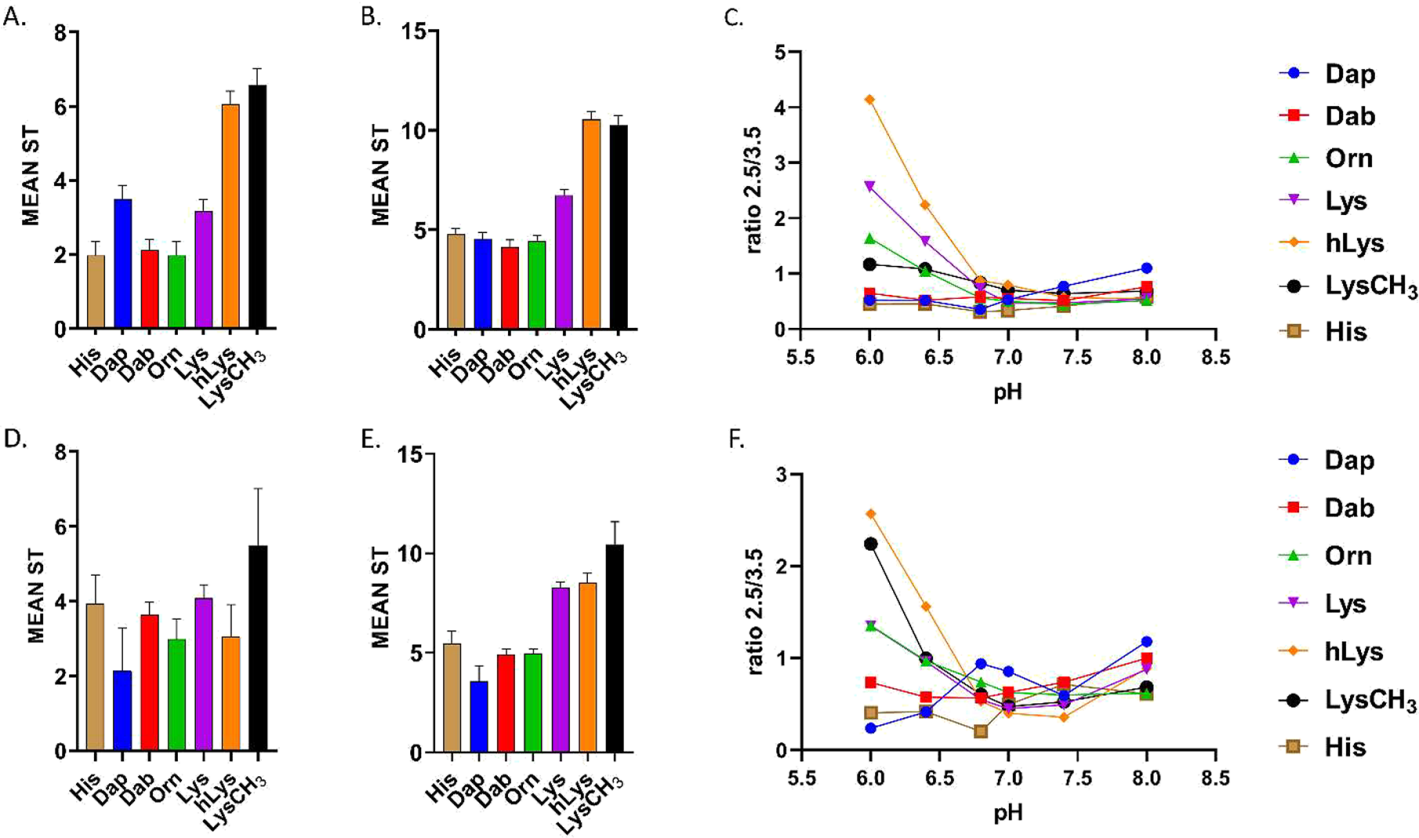
Comparison of the mean ST effects measured at 2.5 ppm and 3.5 ppm (A and B) for the aqueous solutions in PBS (10 mM) of the investigated peptides (*T* = 21 °C, pH = 7.4, saturation power = 1.5 μT, saturation duration = 3 s). (C) Ratiometric curves (2.5/3.5 ppm) for the peptides titrated at several pH values (*T* = 21 °C, pH = 7.4). Comparison of the mean ST effects measured at 2.5 ppm and 3.5 ppm (D and E) for the investigated peptides (*T* = 33 °C, pH = 7.4). (F) Ratiometric curves (2.5/3.5 ppm) for the peptides titrated at several pH values (*T* = 33 °C, pH = 7.4).

By increasing the temperature to 33 °C, a marked enhancement of the CEST contrast was observed for almost all peptides. However, the CEST response between 21 and 33 °C for hLys at 2.5 ppm (Figure 2D) resulted in a noticeable undershoot. This pronounced difference in the amine proton exchange of hLys, compared to that of the analogous protons in the other peptides, is likely due to a faster exchange rate, which results in a broadening of the corresponding resonance. Since the same irradiation power (1.5 μT) was applied, the broadened NH_2_ resonance in hLys is expected to lead to a reduced number of saturated spins transferred to the bulk water signal.

Based on the preliminary screening results (Figure 2), the peptide hLys was selected for detailed characterization. Further tests on hLys devoted to acquiring a detailed characterization of its CEST properties at several conditions of pH, temperature, and concentrations (Figure 3) led us to select it as the candidate of choice for pH-CEST assessment.

**Figure 3:**
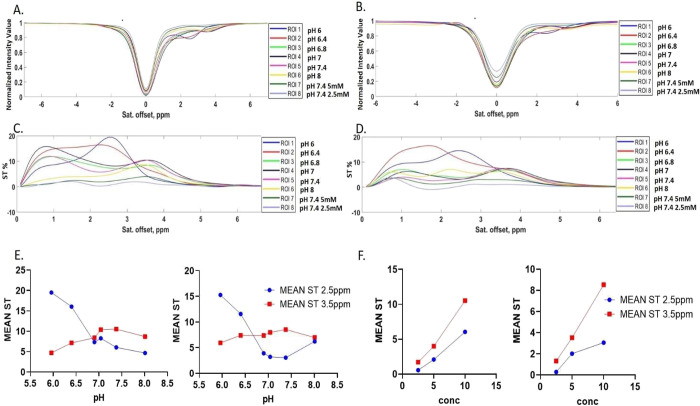
Analysis of the hLys peptide (10 mM) CEST response. Z-spectra and ST curves at *T* = 21 °C (A and C) and at *T* = 33 °C (B and D, at 10 mM concentration). (E) Mean ST (%) as a function of pH for 21 °C (left) and 33 °C (right), respectively. (F) Mean ST as a function of the peptide concentration (2.5, 5, and 10 mM) at *T* = 21 °C (left) and at *T* = 33 °C (right), respectively (saturation power = 1.5 μT and saturation duration = 3 s).

The two pools of mobile protons have a marked and inverse pH dependence (Figure 3E), with the amine group showing a constant decrease of the CEST contrast for pH values from acidic to neutral, whereas the amide group shows a slow but constant increase of the CEST effect upon increasing pH values throughout the investigated pH range. Similar changes, although less pronounced, were observed when the temperature was increased (Figure 3B,D,E). A good and linear CEST contrast dependence on peptide concentration was observed for the range 2.5–10 mM at *T* = 21 °C and *T* = 33 °C (Figure 3E,F, respectively).

### In Vitro Stability of hLys-Containing Peptide in Serum

Chemical and biological stability, along with the absence of toxicity, are crucial requirements for peptides intended for in vivo MRI imaging applications.^58^ These properties ensure that the peptides remain functional and safe, also when dissolved in a complex biological system such as blood serum. One of the major challenges in this context is the high intrinsic proteolytic activity of serum, which continuously processes peptides and proteins, potentially reducing their efficacy and altering their efficiency for the intended application.^59^

The stability of the selected hLys peptide was monitored in human blood serum over time using RP-HPLC. The chromatographic spectra of the peptide degradation products are shown in Figure 4. Remarkably, even after 8 h of incubation at 37 °C, the chromatogram revealed that the peak at *t*_R_ = 4.6 min, corresponding to the intact hLys peptide, remained the only visible peak throughout the chromatographic run. This finding strongly indicates the excellent stability of the peptide in human serum, as no significant degradation products were detected during the observation period. These results suggest that the hLys-containing peptide has an outstanding resistance to proteolytic cleavage, making it a stable probe for in vivo MRI applications. Such stability not only supports its potential as an imaging agent but also minimizes the risk of unintended biological interactions or possible toxic effects resulting from its degradation products.

**Figure 4:**
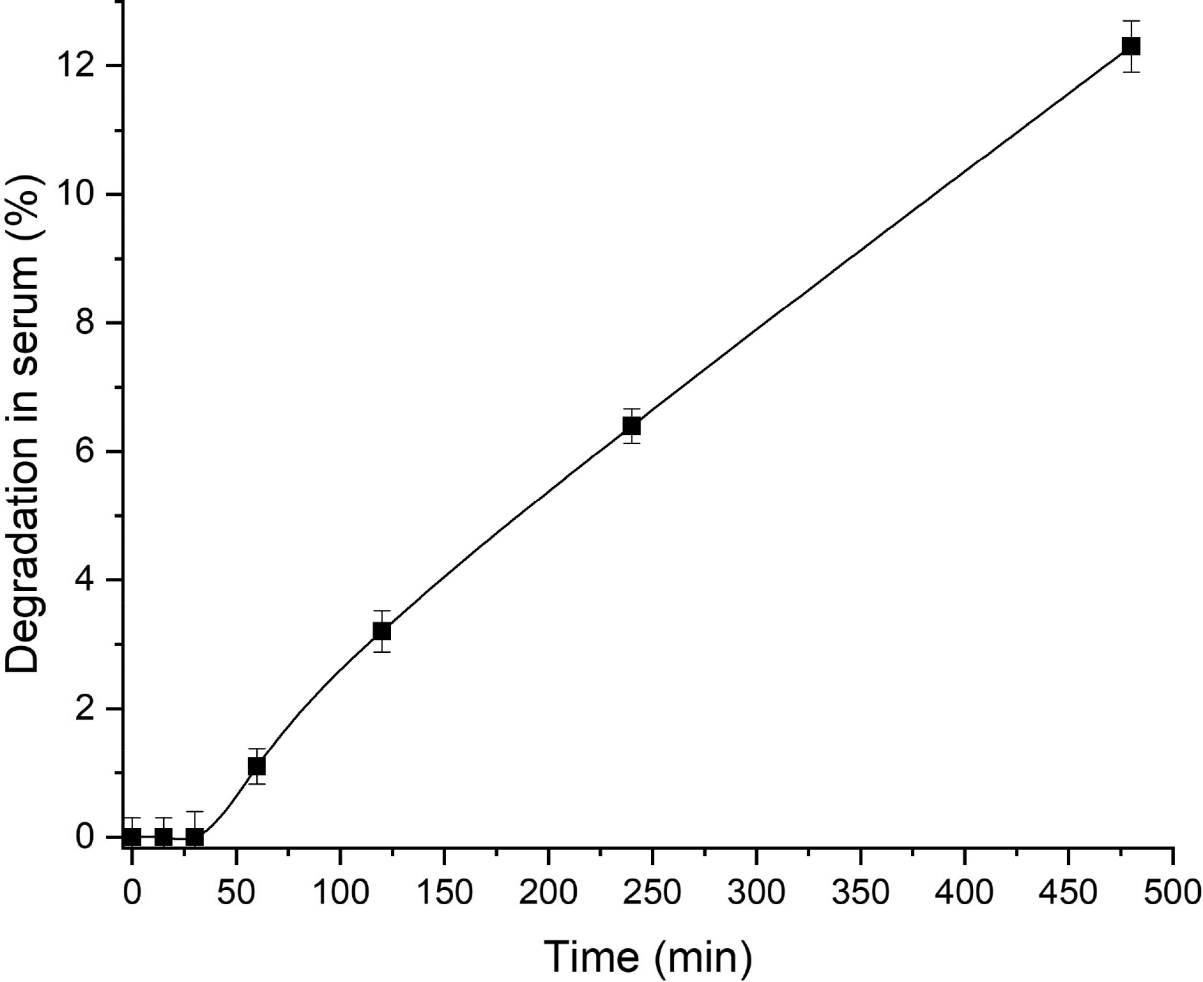
Stability of hLys in human serum. Percentage of degraded peptide over 8 h, data shown as mean ± SD from experiment performed in triplicate.

Indeed, as illustrated in Figure 4, which corresponds to the analysis of the percentage of intact peptide calculated by peak integration, the hLys-containing peptide remains completely unchanged during the first 30 min of incubation. After 8 h, approximately 87.5% of the peptide remains, suggesting a relatively good stability in the serum environment.

At the 28-h time point, an aliquot of the peptide solution was analyzed via LC–MS to gain further insights into its fate. The ESI spectrum (Figure S2) showed a peak at 473.4 *m*/*z*, corresponding to the intact peptide, and two additional peaks at 331.2 and 275.8 *m*/*z*, which correspond to the degradation fragments resulting from the removal of the hLys and hLys-Gly moieties, respectively.

The high stability of this peptide in human serum is likely associated with the incorporation of the unnatural lysine analogue, hLys. The use of such an analogue is a known strategy to protect bioactive peptides from enzymatic degradation, as it can resist cleavage by proteases typically present in blood serum. In fact, several studies have demonstrated that the incorporation of unnatural amino acids or other synthetic modifications can significantly improve the stability and bioavailability of peptides, thereby enhancing their potential for therapeutic and diagnostic applications.^60,61^

These findings further support the potential of the hLys peptide as a stable and effective probe for in vivo applications, where resistance to proteolytic degradation is essential for maintaining peptide function and ensuring a reliable imaging performance.

### In Vivo Performance of the hLys-Containing Peptide as CEST Agent

The marked CEST contrast properties and excellent pH responsiveness of the hLys peptide prompted us to assess its in vivo performance in a murine tumor model. Following i.v. injection of hLys at a dose of 1 mmol/kg b.w., a marked CEST contrast was observed throughout the whole tumor region (Figure 5A), with a mean ΔST contrast higher than 5% at both frequency offsets of 2.5 and 3.5 ppm (Figure 5B). The peptide showed a stable and long-lasting accumulation within the tumor for at least 1 h (Figure 5B).

**Figure 5:**
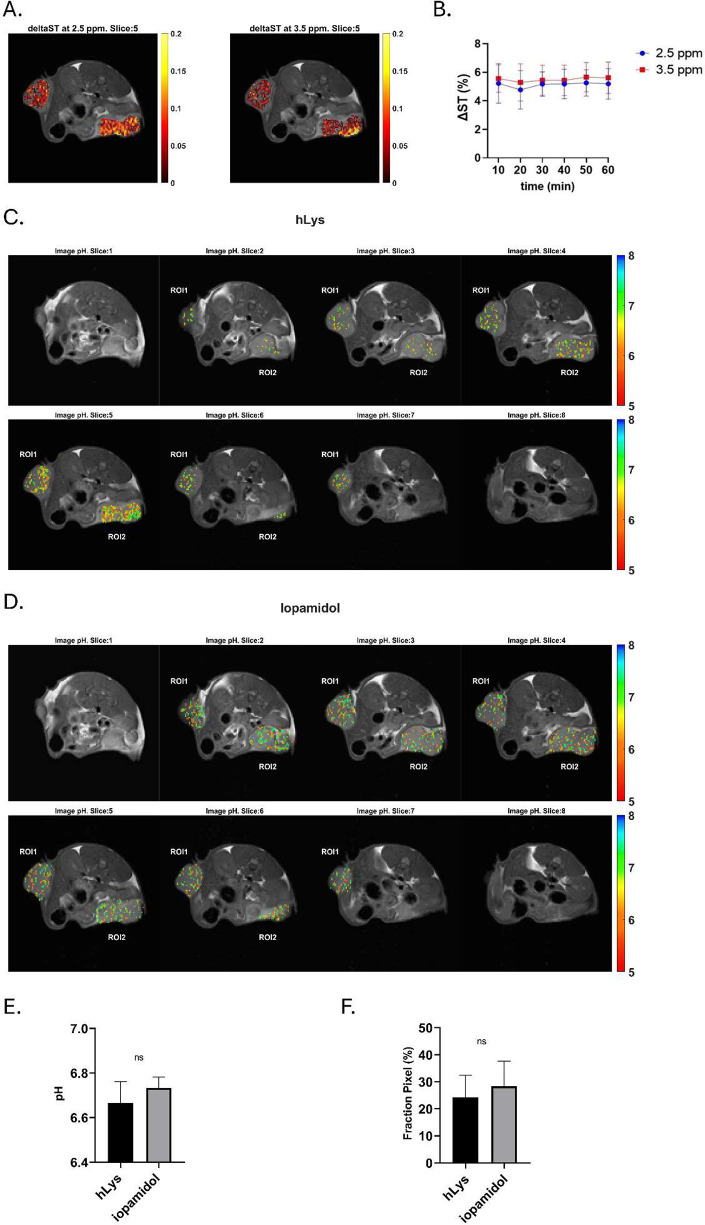
(A) CEST contrast ΔST% maps at 2.5 ppm (left) and at 3.5 ppm (right) in a representative tumor-bearing mouse on both flanks upon i.v. injection of hLys at the 1-mmol/kg dose. (B) Time evolution of the CEST contrast ΔST% up to 60 min upon i.v. injection of hLys for both pools. (C) Comparison of tumor extracellular pH maps calculated following consecutive i.v. injection of hLys at 1 mmol/kg (ROI1 mean pH of 6.6; ROI2 mean pH of 6.56) and (D) of iopamidol at 10 mmol/kg (ROI1 mean pH of 6.7; ROI2 mean pH of 6.66). (E) Comparison of the average extracellular tumor pH values between iopamidol and hLys (*n* = 6 tumors). (F) Comparison of the percentage of extravasated contrast agent on the whole tumor region (extravasation fraction).

On the basis of the in vitro results, we measured tissue pH by taking the ratio of the CEST contrast measured at the two frequency offsets of 2.5 and 3.5 ppm. Tumor pH measured by the hLys-containing peptide showed a marked acidic tumor (Figure 5C), with average pH values of ca. 6.6–6.7 (Figure 5E). Next, the extracellular tumor pH map obtained upon hLys injection was compared with the corresponding pH readout provided by the well-established pH-responsive CA, iopamidol, on the same tumor-bearing mice. In the same mice, during the same MRI session, tumor pH maps obtained upon iopamidol injection showed an analogously marked tumor acidity (Figure 5D), with tumor pH values not statistically different from those provided by the hLys-containing peptide (Figure 5E). Further studies are needed to assess the pH accuracy at the voxel level, in addition to the whole tumor volume, but these require the simultaneous injection of two pH-responsive CAs that could be individually detected. Both pH-responsive CAs showed a moderate accumulation within the tumor volume, with comparable extravasation fractions, likely reflecting a modest tumor vascularization/permeability (Figure 5F).

## Conclusions

In this study, the CEST properties of a group of pentapeptides were evaluated in view of their application as MRI-CEST pH reporters. The modulation of the side chain length using lysine analogues with different aliphatic chain lengths allowed us to explore how these structural modifications affect the proton exchange rates and the overall pH-CEST performance. Our findings provide useful insights into the relationship between molecular structure and CEST efficiency, with implications for the design of more effective peptide-based CAs for biomedical imaging. Among all the synthesized peptides, including one containing His as a side chain exchanging group, the hLys-containing peptide showed the highest pH sensitivity in the 6.0–8.0 range, thanks to the improved CEST response at 2.5 and 3.5 ppm. In vivo MRI-CEST on a murine tumor model confirmed its ability to accurately map acidic tumor pH values. The obtained results were fully consistent with those obtained on the same animals by using the well-established pH-responsive agent iopamidol, with no statistically different average tumor pH values. However, in several pixels inside the tumor region, it was not possible to provide a pH measurement because of a limited accumulation of both pH-responsive CAs, likely reflecting a modest tumor vascularization or permeability. Potential clinical translation of this approach could be limited by the overall acquisition time needed for sampling all the offsets and for covering all the tumor volume, although fast CEST acquisition schemes have already been implemented on clinical scanners.^62–64^ Interestingly, the pH tumor map was obtained by administering a dose of hLys-containing peptide, whose weight is about 8-fold less than that used in the case of iopamidol. These results, together with the peptide’s excellent chemical and biological stability, highlight the potential of the hLys-containing peptide for future MRI-based diagnostic applications, particularly in tumor imaging and in personalized medicine.

## Supplementary Material



## Notes

Figure S1, ESI mass spectra of (A) Dap; (B) Dab; (C) Orn; (D) Lys; (E) hLys; (F) LysCH3; (G) His peptides; Figure S2, ESI mass spectra of an aliquot of hLys peptide after 24 h of incubation with human serum at 37 °C under gentle shaking; and Figure S3, pH calibration curve for hLys (PDF)

## Abbreviations

APT: amide proton transfer
ATCC: American type culture collection
CEST: chemical exchange saturation transfer
CT: computed tomography
CAs: contrast agents
ESI: electrospray ionization source
ECM: extracellular matrix
FBS: fetal bovine serum
FDA: food and drug administration
LC–MS: liquid chromatography–mass spectrometry
MRI: magnetic resonance imaging
PBS: phosphate-buffered saline
RP-HPLC: reversed-phase high-performance liquid chromatography
SPPS: solid phase peptide synthesis
ST: saturation transfer
