# Supplementary material for: Peptide-Based pH-Responsive MRI-CEST Agents: In Vivo Comparison between a Selected Pentapeptide and the Established Iopamidol Reference in Tumor pH Mapping Ability
Source: Chem Biomed Imaging. 2026 Jan 1;4(6):1076–86. doi: 10.1021/cbmi.5c00150 (PMC13291949; doi:10.1021/cbmi.5c00150)
Supplement: Supplementary file 1 [file im5c00150_si_001.pdf]

Supporting information

Peptide-based pH responsive MRI-CEST agents. *In vivo* comparison between a selected pentapeptide and the established iopamidol reference in tumor pH mapping ability

Enrico Gallo,[a] Antonella Carella,[b] Francesco Gammaraccio,[b] Marco Salvatore,[a] Antonella Accardo,[c] Dario Livio Longo,[b] and Silvio Aime*[a]*

[a] Dr. E. Gallo, Prof. M. Salvatore, Prof. S. Aime

IRCCS SYNLAB SDN

via G. Ferraris 144, 80146 Napoli, Italy.

E-mail: enrico.gallo@synlab.it; silvio.aime@synlab.it

[b] Dr. A. Carella, Dr. F. Gammaraccio, Dr. D.L. Longo

Consiglio Nazionale delle Ricerche, Istituto di Biostrutture e Bioimmagini, Sede Secondaria di Torino.

via Nizza 52, 10126 Torino, Italy.

[c] Prof. A. Accardo

Department of Pharmacy and Interuniversity Research Centre on Bioactive Peptides (CIRPeB) “Carlo Pedone” University of Naples Federico II

via D. Montesano 49, 80131 Napoli, Italy.

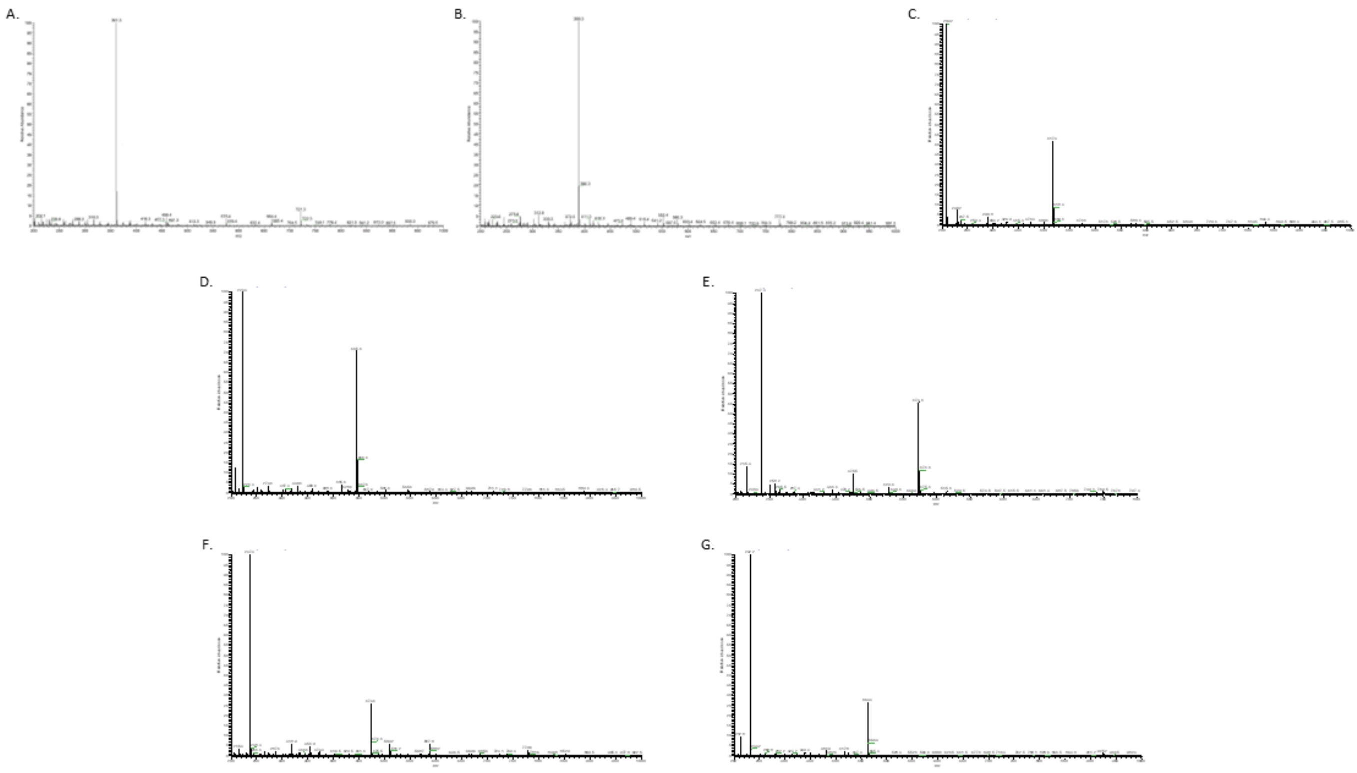

Figure S1. ESI mass spectra of A) Dap; B) Dab; C) Orn; D) Lys; E) hLys; F) LysCH3 G) His peptides.

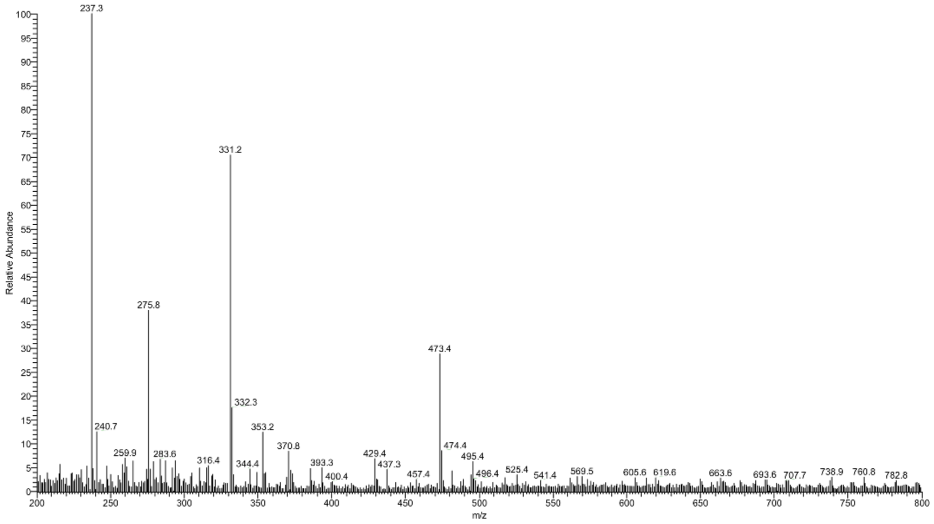

Figure S2. ESI mass spectra of an aliquot of hLys peptide after 24 h of incubation with human serum at 37°C under gentle shaking.

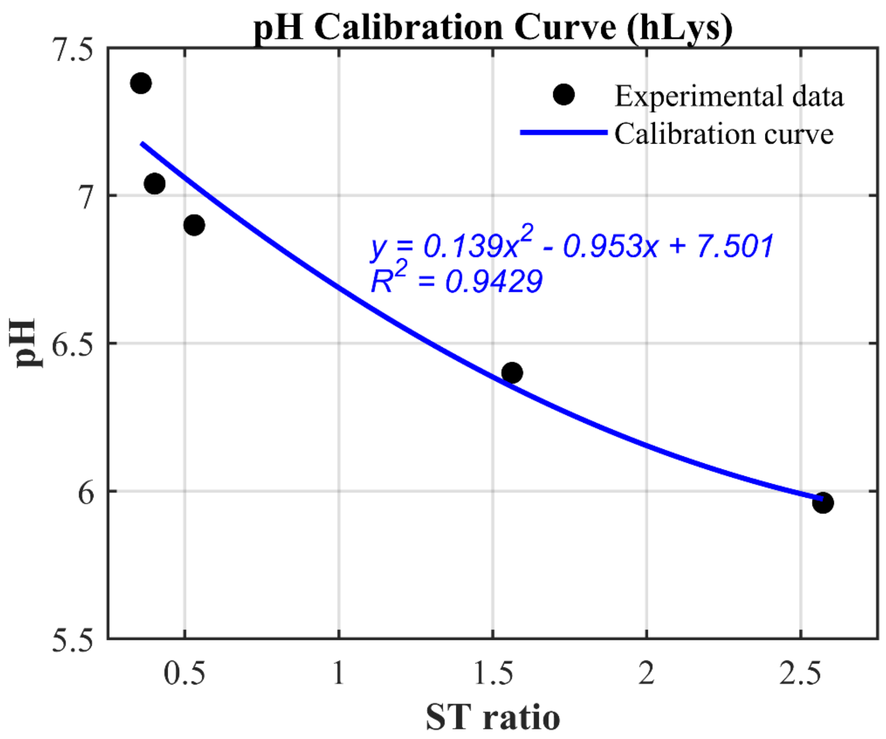

Figure S3. The calibration curve shows the calculated ratiometric values (in black) together with the second-degree polynomial function (in blue) for hLys.
